# The Brustkrebs-Studien.de website for breast cancer patients: User acceptance of a German internet portal offering information on the disease and treatment options, and a clinical trials matching service

**DOI:** 10.1186/1471-2407-10-663

**Published:** 2010-12-02

**Authors:** Markus Wallwiener, Christian W Wallwiener, Sara Y Brucker, Andreas D Hartkopf, Tanja N Fehm, Julia K Kansy

**Affiliations:** 1 The Heidelberg Breast Centre, Department of Obstetrics and Gynaecology, Heidelberg University Hospital, Voßstr. 9, D-69115 Heidelberg, Germany; 2 The Baden-Württemberg Institute for Women's Health, University of Tübingen, Calwerstr. 7, D-72076 Tübingen, Germany; 3 Department of Obstetrics and Gynaecology, Tübingen University Hospital, University of Tübingen, Calwerstr. 7, D-72076 Tübingen, Germany; 4 Department of Oral and Maxillofacial Surgery, Basel University Hospital, Spitalstr. 21, CH-4031 Basel, Switzerland

## Abstract

**Background:**

The internet portal http://www.brustkrebs-studien.de (BKS) was launched in 2000 by the German Society of Senology (DGS) and the Baden-Württemberg Institute for Women's Health (IFG) to provide expert-written information on breast cancer online and to encourage and facilitate the participation of breast cancer patients in clinical trials. We describe the development of BKS and its applications, and report on website statistics and user acceptance.

**Methods:**

Existing registries, including ClinicalTrials.gov, were analysed before we designed BKS, which combines a trial registry, a knowledge portal, and an online second opinion service. An advisory board guided the process. Log files and patient enquiries for trial participation and second opinions were analysed. A two-week user satisfaction survey was conducted online.

**Results:**

During 10/2005-06/2010, the portal attracted 702,655 visitors, generating 15,507,454 page views. By 06/2010, the website's active scientific community consisted of 189 investigators and physicians, and the registry covered 163 clinical trial protocols. In 2009, 143 patients requested trial enrolment and 119 sought second opinions or individual treatment advice from the expert panel. During the two-week survey in 2008, 5,702 BKS visitors submitted 507 evaluable questionnaires. Portal acceptance was high. Respondents trusted information correctness (80%), welcomed self-matching to clinical trials (79%) and planned to use the portal in the future (76%) and recommend it to others (81%).

**Conclusions:**

BKS is an established and trusted breast cancer information platform offering up-to-date resources and protocols to the growing physician and patient community to encourage participation in clinical trials. Further studies are needed to assess potential increases in trial enrolment by eligibility matching services.

## Background

Breast cancer is the most common female malignancy in Germany and worldwide, and its prevalence is predicted to increase [1]. Growing numbers of clinical trials are being conducted to collect the information needed to provide patients with tailored treatments, and the guidelines for certified breast centres in Germany require that 20% of all treated patients be enrolled in clinical trials [2]. However, trial recruitment is difficult, expensive and time-consuming [3]. In the everyday clinical setting, physicians recruit fewer than half of the potentially eligible patients into clinical trials [4]. Major factors preventing patients from participation have been shown to include lack of interest [3,5], low acceptance of the study treatment, concerns about work-related problems, travel and long treatment periods [6,7]. Physicians fail to recruit patients due to reservations about clinical trials and negative referral policies at certain centres, and their patients' comorbidities, mental state and disabilities [8,9]. In addition, the inclusion and exclusion criteria for trials can vary to such an extent that physicians may not have sufficient knowledge to identify eligible patients [10]. On the other hand, 40-50% of cancer patients already resort to the Internet for healthcare information, and this number is likely to increase dramatically over the next few years [4]. As a result of "patient empowerment", patients have come to expect access to reliable, high quality, patient-centred information on their medical condition and all available treatment options.

To meet these demands, the German Society of Senology (DGS) and the Baden-Württemberg Institute for Women's Health (IFG) in 2000 jointly launched the internet portal http://www.brustkrebs-studien.de (BKS) as a not-for-profit service to breast cancer patients and physicians alike [11,12], offering patients extensive information on the disease, its treatment and study participation, and providing investigators with a registry database for their clinical trials. We here describe how BKS was developed and present the results of the implementation phase as a proof of principle.

## Methods

### Development of the BKS website

#### Design of the BKS platform

Existing breast cancer trial registries such as ClincialTrials.gov (maintained by the National Cancer Institute (NCI)), BreastCancerTrials.org, OncoLink.org and a German clinical trials registry maintained by the German Cancer Society, Studien.de, were reviewed and analysed [13-16]. All features were compared, evaluated and prioritized. These were reviewed by an advisory board of breast cancer experts from the German Society of Senology (DGS) and patient support and advocacy organizations to assist with the development process. The advisory board regularly reviewed drafts of BKS to ensure early detection of usability issues as work progressed.

#### Software implementation

HTML/PHP (Hypertext Markup Language/Hypertext Preprocessor) and MySQL (Structured Query Language) were chosen because they are well-established standards and supported by virtually all web browsers. They also offer the benefits of Open Source Software for maximum scalability and extensibility [17,18]. Security measures implemented included the encryption of data transmitted to and from users via HTTPS/SSL (HyperText Transport Protocol Secure/Secure Sockets Layer) and digital certificates [19]. User friendliness was given the highest priority during the design and development phase, in accordance with the findings of Nielsen [20].

#### Trial registry

A registry of all relevant therapeutic breast cancer trials, including protocols, inclusion and exclusion criteria and recruitment status was designed and implemented as one of the three core BKS resources. The advisory board determined the studies to be included in the registry. The principal investigators were assigned the responsibility for maintaining and updating the information on their trials. All trial entries underwent regular quality assurance reviews by the DGS support team.

#### Breast cancer guidebook

To strengthen the portal as a knowledge hub on breast cancer, the DGS published its patient-centred guidebook on BKS.

#### Expert advice

A service was implemented via which patients could contact an expert panel for second opinions or advice on treatment and participation in clinical trials.

### Evaluation of the BKS website

#### Access statistics

User and hit statistics were obtained by log file analysis using the Webalizer 2.1 [21].

#### User interests, portal acceptance and satisfaction

A two-week online survey was conducted during the 2nd and 3rd week of March 2008. To this end, a questionnaire designed to assess user acceptance and satisfaction was announced and made accessible on the BKS homepage. The questionnaire comprised 29 questions with Likert-type scale answers. The questions addressed topics such as the reasons for visiting the website, information quality, quantity and structure of content, awareness of, and interest in, clinical trial participation, and usability aspects of the portal, e.g. page loading time.

#### Statistical analysis

Descriptive statistics were performed using SPSS software (version 9.0 for Windows; SPSS Inc, Chicago IL, USA) to analyse the information submitted via the online questionnaire.

#### Ethics

This study was noninterventional, and no patient-identifiable data were used in the analyses. Therefore the study did not require ethics committee approval or informed patient consent according to the relevant German laws and regulations.

## Results

### The BKS website

**Figure 1:**
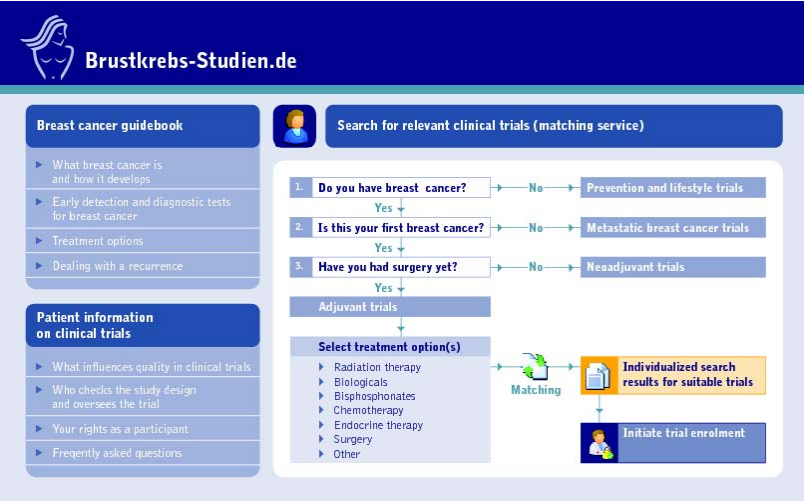
Schematic representation of directly accessible core features on the BKS home page

In 2000, the German Society of Senology (DGS) launched the BKS portal http://www.brustkrebs-studien.de as an information resource and a registry-based matching service for breast cancer patients to facilitate their enrolment in clinical trials. Figure 1 is a schematic representation of the website's core features that can be accessed directly from the home page.

**Figure 2:**
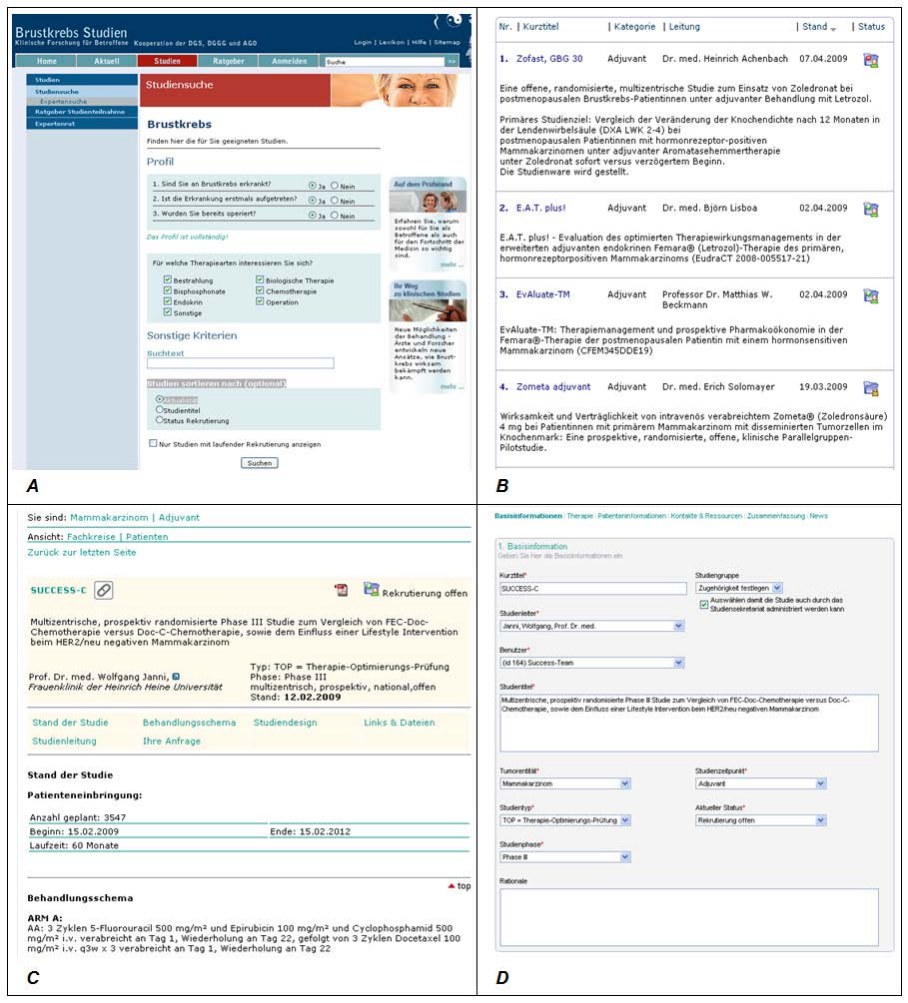
**Screenshots of BKS**. **A**: Patient search; to ensure usability, patients are guided through the wizard using six questions to determine the need for adjuvant or neoadjuvant therapy; **B**: Personalized search results matching the patient's profile; **C**: Example of the study information given for an adjuvant chemotherapy trial, including details of the principal investigator, study design, recruitment status and study regimens; **D**: Principal investigator's input screen for entering details of a clinical trial.

Figure 2 shows exemplary screenshots of the page on the BKS portal where patients can match themselves to relevant clinical trials, pages with search results and study information as well as part of the input mask principal investigators can access to register clinical trials.

The three main components of the BKS portal and their features and functionalities are described in the following.

#### Trial registry

A consistent layout and step-by-step wizards were implemented to help users familiarize themselves rapidly with the system. Investigators receive immediate feedback on information validity when entering protocol information, an approach also used in the NCI's ClinicalTrials.gov database [22]. Investigators can securely publish classified trial documents for physicians via DocCheck^® ^authentication [23]. An automated notification system reminds investigators to update trial information and alerts registered patients to new studies as soon as they are entered into the registry database. Potential study centres can easily contact principal investigators via the system to enquire about inclusion of their site in the database.

Patients can search the trial registry for suitable clinical trials via a simplified wizard-based matching service. Three basic screening questions establish the patient's disease and treatment profile (Figure 2, panel A). The search for suitable studies is then based upon individual parameters and refined by specific criteria, such as treatment modalities, anti-cancer drugs, trial status and randomization type. Following the basic search, patients can securely forward their details anonymously to a particular principal investigator for further eligibility testing.

#### Breast cancer guidebook

Written by prominent members of the DGS, this core resource of the BKS portal provides detailed information on the pathogenesis, types, prevalence, early detection and diagnosis of breast cancer, treatment options, follow-up care and support resources, while also including interactive modules, such as an online audio book ("From diagnosis to follow-up care") and 3D video animations. The guidebook is based on the high quality criteria for health related websites [24]. Another section, added to facilitate the provision of basic informed consent, offers information on clinical trial participation, different trial designs and the procedures involved such as randomization and blinding, the quality of trials and participants' rights and obligations.

#### Expert advice

An expert advisory board of leading breast cancer specialists can be contacted via the platform for second opinions and personal treatment recommendations with regard to clinical trials.

### Evaluation of the BKS website

#### Access statistics

**Figure 3:**
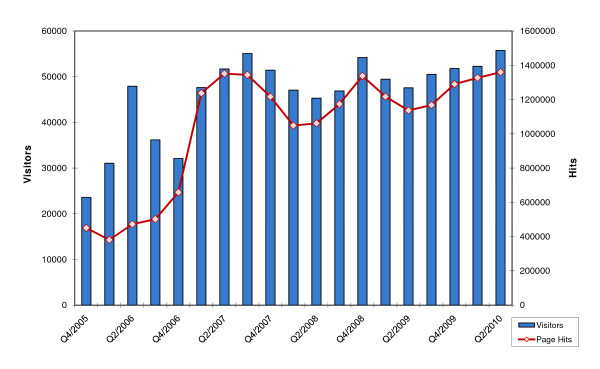
Log file analysis for 1 October 2005 to 30 June 2010

Between 1 October 2005 and 30 June 2010, 702,655 visitors logged onto the portal, generating a total of 15,507,454 page views (quarterly average: 46,183 visitors [range 23,583-55,738]). Figure 3 shows access details from the log file analysis. Mean website visit duration was 3.2 minutes.

#### Patient enquiries for trial participation and second opinions

In 2009, 143 patient enquiries about trial participation were securely forwarded to the principal investigators for eligibility screening. In the same period, 119 patients sought a second opinion or individual treatment advice online via the expert panel.

#### User evaluation of BKS

The portal attracted 5,702 visitors during the two-week online survey period. In total, 568 questionnaires were submitted, of which 507 (89%) were evaluable. Visitors were predominantly patients with breast cancer (67%) or friends and relatives (25%). Most visitors stated that they used the Internet as a source for information about breast cancer (81%), and some regularly forwarded information on the disease to others or exchanged such information (33%).

Most participants found the information on BKS well organized (79%), useful (79%), adequate (63%) and easy to navigate through (87%). The most frequently accessed content in the guidebook was information on the pathogenesis and diagnosis of breast cancer (42%) and treatment options (34%). Most users were confident that the information offered was correct (80%). Pages loaded quickly enough (83%).

More than three-quarters (83%) of respondents called for a discussion forum as an additional feature to the portal. About two-thirds (68%) stated they would be happy to store their medical details online in an anonymous breast cancer record and about three-quarters (74%) were interested in communicating individually with an expert (e.g. for a second opinion and treatment options). Most visitors indicated that they intended to use the portal again in the future (76%) and would recommend it to others (81%).

#### Patient attitudes towards trial participation

Most patients considered it valuable to be able to search for clinical trials themselves (79%), and a majority had already considered trial participation (62%) and previously sought information on participation in clinical trials (54%). The information sources on trial participation that respondents considered useful were the Internet (29%), the patients' physicians (27%) and other patients or support groups (15%).

#### Trial registry

By 30 June 2010, a community of 189 investigators and physicians was contributing to the portal, including all major breast cancer trials groups in Germany, who regularly updated the information on their trials. At that time, the registry covered 163 trials, of which 89 (55%) investigated recurrent disease and 51 (31%) evaluated adjuvant therapy, 15 (9%) neoadjuvant therapy, 4 radiotherapy and 4 surgical treatment (2% each). No trials of preventive interventions had been registered. The 89 (55%) of studies in patients with recurrent disease comprised 27 (30%) with chemotherapy, 8 (9%) with biologicals, 6 (7%) with endocrine treatment, 2 (2%) with radiotherapy and 6 (7%) with other treatment approaches. Of the 51 (31%) adjuvant trials 6 (12%) used chemotherapy, 4 (8%) biologicals, 2 (4%) bisphosphonates, 3 (6%) endocrine treatment, and 1 (2%) other treatments. Table 1 lists the most frequently visited protocols during the 5-month period from 1 August to 12 December 2008.

**Table 1:** Most frequently visited trial protocols between 1 August and 31 December 2008

Study acronym	Full study title	Average weekly hits
SUCCESS	Simultaneous study of gemcitabine-docetaxel combination adjuvant treatment, as well as extended bisphosphonate and surveillance-trial	1,548
GAIN - GBG 33	A study to compare ETC vs. EC-TX and ibandronate vs. observation in patients with node-positive primary breast cancer	1,008
FACE	Comparison trial of letrozole and anastrozole in the adjuvant treatment of postmenopausal women with hormone receptor and node positive breast cancer	488
NATAN	Postoperative use of zoledronic acid in breast cancer patients after neoadjuvant chemotherapy	469

#### Study types in the BKS registry compared with ClinicalTrials.gov (NCI)

An analysis conducted in March 2008 showed that 52% of the BKS trials were in patients with recurrent disease, as opposed to only 2.3% of the NCI trials. The majority (70%) of the NCI trials investigated neoadjuvant therapies (26%), radiotherapy (20%) or surgical treatment (24%).

## Discussion

Over the past few decades, the concept of tailored treatment for breast cancer has led to an ever-increasing need for clinical studies, but patient enrolment has generally been too slow to meet the demands of valid guidelines. As a result of patient empowerment, the demands for reliable, patient-centred information have also grown. The integration of the patient as an active participant in the decision-making process and a partner requiring comprehensive, up-to-date, correct, comprehensible information reflects the changing patient-physician relationship. The Internet has become a key source for patients who wish to be well informed [25], and it has been identified as a powerful vector in increasing trial recruitment rates [4]. It has also been shown that the knowledge- and data-intensive processes of determining patient eligibility can be facilitated by using computerized systems [26]. Here, we describe how the German Society of Senology (DGS) and the Baden-Württemberg Institute for Women's Health (IFG), in collaboration with patient support and advocacy groups, developed and established a trusted online information and communication platform featuring up-to-date trial protocols and resources for the growing physician and patient community. Similar to the NCI's ClinicalTrials.gov, the BKS portal is an official resource backed by a national specialist medical society and aims to serve patients, doctors and researchers alike.

It is unrealistic to assume that physicians can screen every patient for trial eligibility in view of the vast number of clinical trials available today. A large number of potential participants are therefore lost. It has been suggested that only 2-4% of patients with newly diagnosed cancer participate in clinical trials [27]. Although online trial registries seem to be a preferred solution to this dilemma, trend analysis has indicated that it is unlikely that a single trial database would function worldwide, enabling multiple domain-, funder- or country-specific registers to be created [28].

Over the past few years, we have established the BKS website as a highly frequented breast cancer portal with a total of over 700,000 visitors by the middle of 2010. On average, 46,183 visitors accessed the BKS portal per quarter during the period from 1 October 2005 to 30 June 2010.

The true value of the BKS became apparent in 2009, during which year 143 patients contacted investigators via the website to enquire about trial enrolment and 119 requests for second opinions were submitted via the secure e-messaging service. The benefits of e-messaging have been widely discussed in the literature and include decreased numbers of telephone calls [29], unnecessary clinic visits [29-31], improved patient care [32], greater efficiency in the exchange of information between physicians and patients [29] and cost savings [33].

The respondents to our online survey showed a high degree of satisfaction with both the design of the portal and the information provided, and also expressed great trust in the information. The great majority of respondents stated that they would recommend the portal to others and continue using it in the future. Patients have been shown to respond positively to portals backed by a national specialist medical society because they are perceived as more trustworthy [34], and this is a likely reason for the success of our portal, which is officially supported by the German Society of Senology (DGS).

By the middle of 2010, the BKS covered a total of 163 current breast cancer trials, which the advisory board had selected as the most important trials. The active scientific BKS community at the time consisted of 189 physicians and investigators. The majority of trials (55%) focused on the use of chemotherapy in recurrent disease. However, the most visited trials were those investigating adjuvant chemotherapy regimens. This is especially relevant since the majority of trials registered with the NCI during our study period were neoadjuvant studies, possibly reflecting the future focus of research in breast cancer trials.

The success of trial registries depends on a number of factors. One of the main issues in the development of the BKS portal was the need for up-to-date information on trial status, which meant that principal investigators had to take responsibility for updating the information on their trials and removing closed trials from the database. To achieve this, the system was equipped with an automated notification and feedback service to encourage commitment on the part of both the support team and the investigators.

Studies suggest that female patients are generally in favour of clinical trials [35,36], and this is supported by our finding that almost two-thirds of the BKS visitors considered participating in a trial. However, complex trial descriptions and eligibility criteria, the extensive use of medical terminology and expecting patients to determine preferences early on in the registration process act as strong barriers to trial participation [8]. Patients with previous clinical trial experience have fewer concerns than those facing this option for the first time, and recommendations by clinicians play a significant role in their decision [10]. For these reasons, and based on the suggestions of Gillen et al. [22], the emphasis in the development of the BKS portal was placed on patient-friendly versions of trial protocols embedded into relevant patient information and supported by leading breast cancer experts. Our user analysis showed that most patients appreciated being able to search for suitable trials themselves, and that patients relied on the Internet, and not their doctor, for information on trials. To concentrate on patient-friendliness and provide high-quality content was therefore the right approach. To ensure that patients' needs were addressed appropriately, all development work was, and continues to be, conducted in close collaboration with patient support and advocacy organizations, notably "Frauenselbsthilfe nach Krebs" (Women's Self-Help after Cancer) and "Mammazone".

A further trend observed in our analysis is that patients are interested in online personal health records, which is consistent with earlier reports [10]. Although this trend has been known for some years, no approach to patient disease management has been successfully adopted at a national level so far. The BreastCancerTrials.org service [13], however, offers a promising approach. It functions as an online patient breast cancer record combined with a matching service to suitable trials. OnkoLink is a similar portal but is not breast cancer specific [37]. A matching service for trial eligibility based on the US National Cancer Institute's Physician Data Query (PDQ) database was introduced by Ohno-Machado et al. [38]. Not only has it been shown that computer-based, automated screening is superior to physician-based individual screening [39], but also that an online system gives patients more information and more time to decide, and also increases their confidence in their decision-making [40]. Before the BKS portal was initiated, no such approach had been pursued in Germany for breast cancer - a shortcoming that has now been addressed.

One of the limitations of online services for study recruitment in this population is that female breast cancer most frequently occurs after the sixth decade of life, at which age women may be less likely to use online services. Moreover, minorities tend to be underserved with regard to Internet access, especially in rural areas [41]. Clinicians should therefore bear in mind that clinical trial recruitment via the Internet may introduce bias [42]. Thus, potential selection bias represented another limitation to our user analysis. Further limitations included the relatively small sample size, the short period during which the survey was conducted, and that it is unclear whether the participants were representative of the general population, e.g. with regard to age distribution. Despite these limitations, we are confident that the largely very positive answers from the respondents confirmed our approach to launch the BKS portal and that they provided useful information for further enhancements to the service.

Web-based clinical trial portals have great potential as a tool for physicians to manage an increasing number of clinical studies and for patients to access accurate and up-to-date information. Developed by a team physicians and investigators, patient support and advocacy groups and the German Society of Senology, the BKS website already effectively meets the needs of all types of users with regard to data security, privacy and ease of use. A conceivable future refinement of our system will include the creation of pools of eligible patients who have expressed interest in clinical trial participation, undergone preliminary screening and given basic consent. Work is in progress to expand the service by introducing a tool for cross-matching patient demographic and clinical data with trial inclusion and exclusion criteria. Integration of such services into hospital information systems would greatly facilitate preliminary screening and mean that a web-based tool of this kind could be integrated into everyday clinical practice. Most importantly, outcome research is now needed to determine whether such tools actually result in improved recruitment to clinical studies.

## Conclusions

We conclude that http://www.brustkrebs-studien.de is an established and trusted interactive platform providing information on breast cancer for patients and physicians alike. With the aim of encouraging participation in clinical trials, it offers the growing community of patients and physicians seeking information on the internet a range of up-to-date resources including expert-written content on the disease, current treatment options and clinical trial protocols. Further studies are needed and being undertaken to assess potential increases in trial enrolment by eligibility matching services.

## Abbreviations

**BKS**: http://www.brustkrebs-studien.de;** DGS**: German Society of Senology (Deutsche Gesellschaft für Senologie); **HTML**: Hypertext Markup Language; **HTTPS/SSL**: HyperText Transport Protocol Secure/Secure Sockets Layer; **IFG**: Baden-Württemberg Institute for Women's Health (Institut für Frauengesundheit Baden-Württemberg); **NCI**: National Cancer Institute; **PHP**: Hypertext Preprocessor

## Competing interests

The authors declare that they have no competing interests.

## Authors' contributions

MW participated in the development of the website described, conceived of the present study and its design, participated in data analysis and drafted and finalised the manuscript. CWW participated in the development of the website described, contributed to data collection and analysis and reviewed the draft manuscript. SYB, AH, TNF and JKK participated in the development of the website described, contributed to data collection and analysis and reviewed the draft manuscript. All authors read and approved the final manuscript.

## Pre-publication history

The pre-publication history for this paper can be accessed here:

http://www.biomedcentral.com/1471-2407/10/663/prepub
